# Evaluation of ABD-Linked RM26 Conjugates for GRPR-Targeted Drug Delivery

**DOI:** 10.1021/acsomega.4c00489

**Published:** 2024-08-15

**Authors:** Ábel Nagy, Ayman Abouzayed, Panagiotis Kanellopoulos, Fredrika Landmark, Ekaterina Bezverkhniaia, Vladimir Tolmachev, Anna Orlova, Amelie Eriksson Karlström

**Affiliations:** † Department of Protein Science, School of Engineering Sciences in Chemistry, Biotechnology and Health, KTH Royal Institute of Technology, AlbaNova University Center, 106 91 Stockholm, Sweden; ‡ Department of Medicinal Chemistry, Uppsala University, 752 37 Uppsala, Sweden; § Research Centrum for Oncotheranostics, Research School of Chemistry and Applied Biomedical Sciences, Tomsk Polytechnic University, 634009 Tomsk, Russia; ⊥ Department of Immunology, Genetics and Pathology, Uppsala University, 752 37 Uppsala, Sweden; # Science for Life Laboratory, Uppsala University, 752 37 Uppsala, Sweden

## Abstract

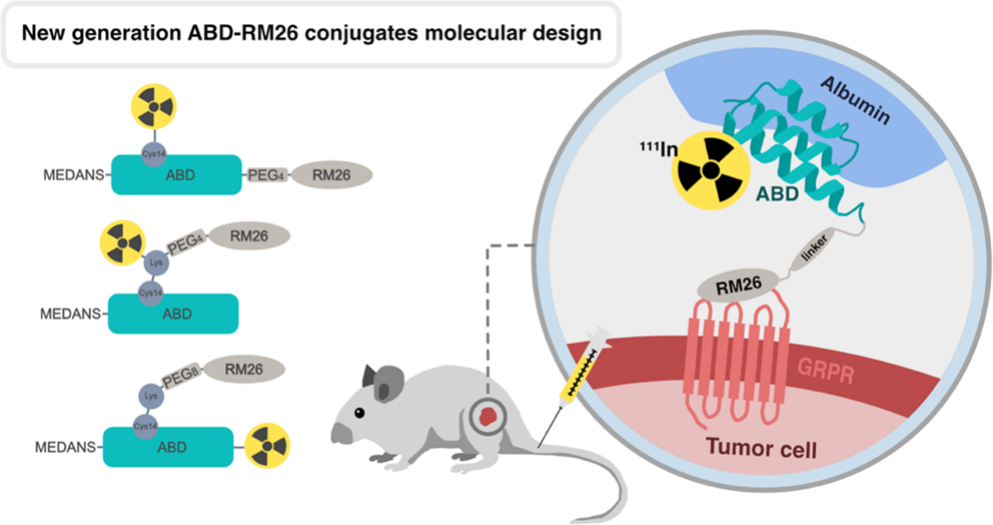

Targeting the gastrin-releasing peptide receptor (GRPR) with the bombesin analogue RM26, a 9 aa peptide, has been a promising strategy for cancer theranostics, with recent success in radionuclide imaging of prostate cancer. However, therapeutic application of the short peptide RM26 would require a longer half-life to prevent fast clearance from the circulation. Conjugation to an albumin-binding domain (ABD) is a viable strategy to extend the *in vivo* half-life of peptides and proteins. We previously reported an ABD-fused RM26 peptide targeting GRPR (ABD-RM26 Gen 1) that showed prolonged and stable tumor uptake over 144 h; however, the observed high kidney uptake indicated that the conjugate’s binding to albumin was reduced and that this could be an obstacle for its use as a delivery system for targeted therapy, especially for radiotherapy. Here, we have designed, produced, and preclinically evaluated a series of novel ABD-RM26 conjugates with the aim of improving the conjugate’s binding to albumin and decreasing the kidney uptake. We developed three second-generation constructs with varying formats, differing in the relative positions of the targeting moieties and the radionuclide chelator. The produced conjugates were radiolabeled with indium-111 and evaluated *in vitro* and *in vivo*. All constructs displayed improved biophysical characteristics, biodistribution, and lower kidney uptake compared to previously reported first-generation molecules. The ABD-RM26 Gen 2A conjugate showed the best biodistribution profile with a nearly 6-fold reduction in kidney uptake. However, the ABD-RM26 Gen 2A conjugate’s binding to GRPR was compromised. This conjugate’s assembly of albumin- and GRPR-binding moieties might be used for further development of drug conjugates for targeted therapy/radiotherapy of GRPR-expressing cancers.

## 1 Introduction

Prostate cancer is one of the leading causes of cancer-related death among men worldwide and its effective diagnosis and treatment remain a challenge.^1^ Identifying and targeting prostate-specific antigens for therapy is a promising strategy to improve the prognosis of the disease. A widely studied target for both diagnostic and therapeutic purposes is the gastrin-releasing peptide receptor (GRPR), which is a member of the bombesin receptor family. This G protein-coupled receptor regulates several functions such as gastric acid and pancreatic secretion and is found to be expressed in the pancreas and stomach.^2^ However, GRPR overexpression has been identified in various malignancies, such as prostate, breast, ovarian, and colon cancer.^3−5^ Overexpression of the receptor is more prominent in the earlier stages of prostate cancer and can be observed in lymph node and bone metastases,^6^ making it a clinically relevant target for cancer theranostics and particularly for oligometastatic prostate cancer.

Clinical translation of natural peptide ligands and their analogues targeting overexpressed receptors such as GRPR, prostate-specific membrane antigen (PSMA), or somatostatin receptors for targeted cancer therapy has gained a lot of momentum in the past years.^7,8^ The natural peptide ligand of GRPR, bombesin (BBN), and its agonistic and antagonistic analogues, including RM26 (d-Phe-Gln-Trp-Ala-Val-Gly-His-Sta-Leu-NH_2_), have been extensively studied as theranostic candidates. In contrast to BBN, RM26 is an antagonist, which was shown to be more favorable for GRPR targeting.^9^ Studies showed that antagonistic receptor binding resulted in better pharmacokinetics, avoidance of receptor downregulation, and activation of physiological responses.^10−12^ Due to the high-affinity binding to the receptor and fast blood clearance, the most attractive application of RM26 has been as a radiotracer for diagnostic purposes. RM26 linked to a macrocyclic chelator with a PEG linker has shown favorable pharmacokinetics in various formats.^13−16^ Most recently, the PET and SPECT tracers [^68^Ga]Ga-NOTA-PEG_3_-RM26 and [^99m^Tc]Tc-maSSS-PEG_2_-RM26 were evaluated clinically and found to be safe and well tolerated in prostate and breast cancer patients.^17−19^ Following their success as imaging agents, bombesin analogues are also gaining attention for therapeutic application. The antagonistic analogues labeled with the therapeutic radionuclides Lu-177 and Y-90 demonstrated significant therapeutic effect in a preclinical model.^20−22^ The analogue [^177^Lu]Lu-RM2 was clinically studied for the treatment of metastatic prostate cancer and was confirmed to be safe, and no adverse effects were observed.^23^

The main challenge of short therapeutic peptides is their fast clearance from the blood circulation, requiring multiple and frequent injections for efficacious treatment. For example, the estimation of dosimetry for radiotherapy in a murine model demonstrated that 6 injections of RM26 labeled with Lu-177 within 2 weeks are required for effective treatment.^20^ There are several strategies developed to extend the circulatory half-life of such therapeutics, including Fc- or serum albumin-fusion, PEGylation, or conjugation to albumin-binding domain (ABD).^24,25^ Binding to human serum albumin (HSA) through ABD increases the protein’s size as well as allows FcRn-mediated recycling of the whole complex.^26^ ABD originates from the streptococcal protein G and has been engineered to bind HSA with femtomolar affinity.^27^ This engineered variant, ABD035, has been used to prolong the half-life of several small peptides and scaffold proteins.^28^ Due to its bacterial origin, using ABD in human applications might be a concern in terms of immunogenicity. However, further engineering to reduce immunogenicity by removing potential T-cell epitopes has been carried out,^29^ and in clinical studies, an ABD-fused anti-IL17A affibody used for the treatment of psoriasis was demonstrated to be well tolerated by patients.^30^

In our previous study, we reported an ABD-fused RM26 conjugate, DOTA-ABD-RM26, that structurally resembled the previously reported glucagon-like peptide 1 (GLP-1)-targeting conjugate GLP-1-ABD.^31^ The GLP-1-ABD conjugate preserved both a strong affinity for albumin and the ability to stimulate insulin release. However, the properties of GLP-1-ABD were not studied *in vivo*. The ABD-RM26 conjugate, when labeled with In-111, displayed significantly higher blood retention and prolonged circulatory half-life *in vivo* compared to its parental peptide, resulting in stable tumor uptake of the radiolabeled conjugate even after several days.^32^ The biodistribution of ABD-RM26 was similar to the biodistribution of ABD-fused scaffold-based drug conjugates and suggested that this GRPR-targeting conjugate could be used for drug/toxin delivery.^33−35^ However, undesired elevated kidney uptake could prevent its further application for radionuclide therapy; activity uptake was 2–3-fold higher than for the ABD-fused targeting scaffold proteins that demonstrated efficacy for targeted radiotherapy in a preclinical model.^36−38^

The optimization of the agents for targeted therapy, including radiotherapy, should take into account several aspects of their biological properties. This includes, but is not limited to, the efficient development of the cytotoxic moiety to the target, homogeneous distribution of the agent in the targeted tissue, strong binding of the agent to the target and long retention in the targeted tissue, and low accumulation in healthy nontargeted tissue.^39^ The ABD-conjugated peptides might satisfy many of these requirements. The ABD-mediated prolonged blood circulation should increase their bioavailability and thus increase accumulation in targeted tissue.^40^ The size of the albumin-ABD adduct is smaller than the size of intact antibodies, which should facilitate their tissue penetration and homogeneous distribution in the targeted tissue. The ABD-fused proteins tend to have low absorption in excretory organs.^36,40^ The main limiting organs for targeted therapy are excretory organs, liver and kidneys, organs with endogenous target expression, and bone marrow in case of radiotherapy.^39^ The effective radiotherapeutic agent should deliver at least a 25-fold higher dose to the tumor than to the bone marrow and a 2-fold higher dose than to the kidneys to reach therapeutic effect in tumor without hematological and/or renal toxicity.^41^ The absence of unspecific binding of the targeting agent to blood proteins should help to avoid additional doses to the bone marrow, while low reabsorption of the agent in kidneys should reduce renal toxicity. We hypothesized that the unfavorably high kidney uptake was due to the observed lower thermal stability and helicity of the ABD in the conjugate compared with the parental ABD molecule. Having the conjugate present in a partially unfolded state *in vivo* could compromise the interaction with HSA, which could result in an unfavorable biodistribution profile. These observations prompted us to pursue further engineering of ABD-RM26 conjugates with a higher thermal stability and improved *in vivo* properties.

The molecular design of a targeting agent can have a strong influence on the *in vivo* properties and biodistribution. Depending on the sequence and relative positions of the functional domains, and the composition of linkers or termini, the interaction of molecules with their targets can be affected.^42,43^ Therefore, careful optimization of the molecular design is important for the development of therapeutic molecules. Combining the GRPR-targeting RM26 peptide with ABD, a half-life extension moiety, and the macrocyclic chelator 1,4,7,10-tetraazacyclododecane-1,4,7,10-tetraacetic acid (DOTA) for radiolabeling within one complex molecule, while preserving each of these functionalities, presents a great engineering challenge. Considerations for selecting an ideal position for RM26 cross-linking and DOTA conjugation were based on results from previous studies. Position 14 in helix 1 of ABD has been shown to be a suitable location for the attachment of other molecules without compromising HSA binding.^31^ This position can be utilized for the attachment of RM26 or a DOTA chelator to ABD. For orthogonal labeling, the C-terminus of the ABD can be extended with a short peptide sequence, making it a substrate for enzymatic cross-linking using Sortase A transpeptidase.^44^ Furthermore, the composition of the N-terminus has been shown to contribute to the biodistribution profile as well as the stability of the molecule in similar scaffold molecules.^45,46^

In this study, we have designed, produced, and evaluated three second-generation ABD-RM26-DOTA conjugates, with the overall aim of improving the biodistribution profile while maintaining a prolonged half-life and tumor specificity. Our results reveal that the relative positions of the protein-binding domains, RM26 and ABD, and the radiometal-DOTA complex have significant effects on biodistribution and tumor uptake.

## 2 Results

### 2.1 Molecular Design

**Figure 1:**
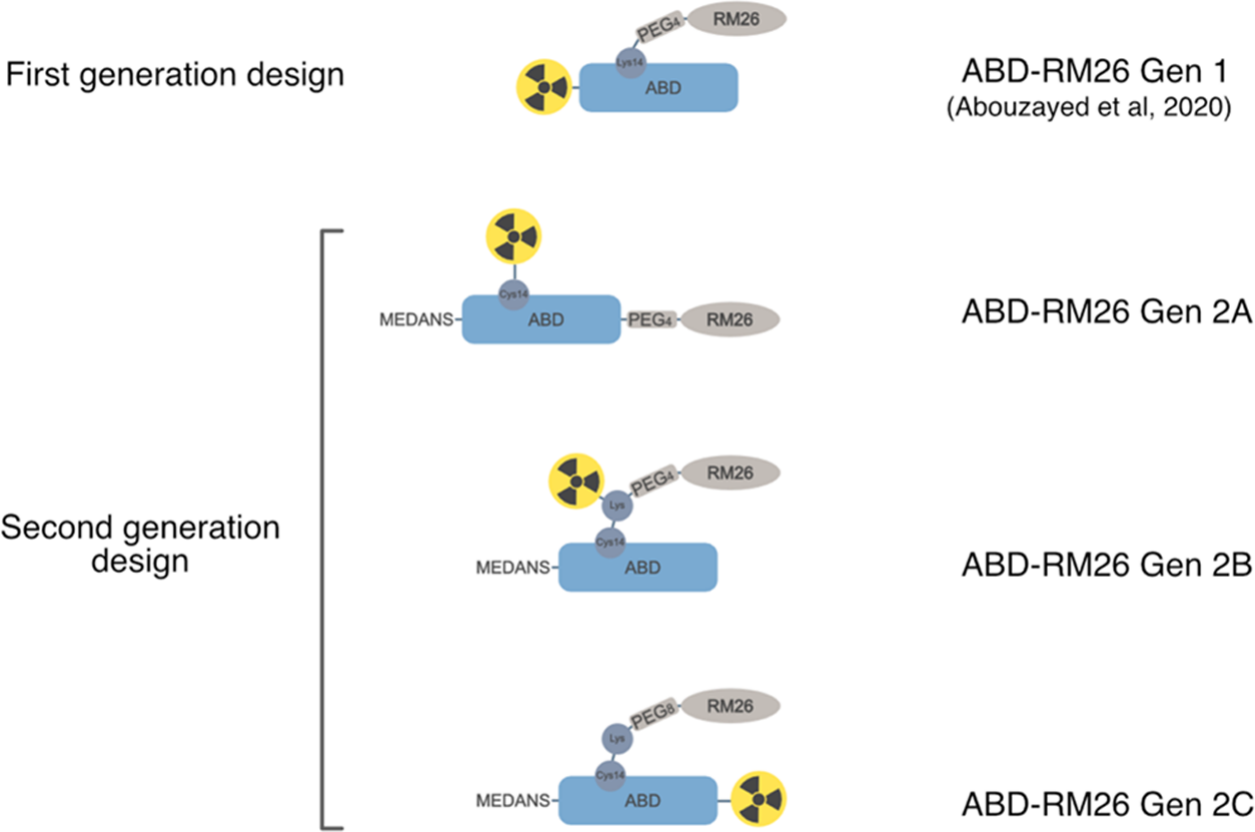
Schematic representation of the ABD-RM26 s-generation molecular designs. ABD: albumin-binding domain. PEG_4_: 15-amino-4,7,10,13-tetraoxapentadecanoic acid. DOTA: 1,4,7,10-tetraazacyclododecane-1,4,7,10-tetraacetic acid.

Three second-generation ABD-RM26 conjugates were designed by combining the albumin-binding domain (ABD), the RM26 peptide, and the DOTA chelator in different formats. A schematic representation of the molecular constructs can be seen in Figure 1, and ABD and the RM26 peptide sequences produced and synthesized for the corresponding constructs are displayed in Table 1. Our previously reported first-generation conjugate consisted of a fully synthetic ABD-PEG_4_-RM26 molecule, which we opted to replace with a semisynthetic conjugate combining recombinantly produced ABD conjugated to RM26 synthesized by solid-phase peptide synthesis (SPPS). For orthogonal conjugation purposes, a thiol-group-containing cysteine residue was introduced in the sequence of ABD at position 14, and a Sortase A recognition motif (LPETGG) was added to the C-terminus of ABD.

**Table 1:** Peptides and Proteins Used for the Production of Gen 2A, 2B, and 2C ABD-RM26-DOTA Conjugates^a^

name	sequence
ABD	MEDANSLAEAKVLANRELDKYGVSDFYCRLINKAKTVEGVEALK LHILAALPVDGSGSGSLPETGGHHHHHH
RM26 used in Gen 2A	Gly-Gly-Gly-[PEG_4_]-d-Phe-Gln-Trp-Ala-Val-Gly-His-Sta-Leu-NH_2_
RM26 used in Gen 2B	[DOTA]-Lys(AcCl)-[PEG_4_]-d-Phe-Gln-Trp-Ala-Val-Gly-His-Sta-Leu-NH_2_
RM26 used in Gen 2C	Lys(AcCl)-[PEG_4_]-[PEG_4_]-d-Phe-Gln-Trp-Ala-Val-Gly-His-Sta-Leu-NH_2_

a RM26 amino acid sequences are shown in three-letter code, and the ABD sequence is shown in one-letter code. PEG4: 15-amino-4,7,10,13-tetraoxapentadecanoic acid. DOTA: 1,4,7,10-tetraazacyclododecane-1,4,7,10-tetraacetic acid.

In the second-generation conjugate 2A (ABD-RM26 Gen 2A), the RM26 peptide was enzymatically cross-linked to the C-terminus of ABD in a Sortase A-mediated reaction. As a spacer between the two domains, a PEG_4_ (15-amino-4,7,10,13-tetraoxapentadecanoic acid) linker was added to introduce more flexibility and accessibility for GRPR receptor binding of RM26 and HSA binding of ABD. The DOTA chelator was coupled to the thiol group of Cys14 in ABD.

In the second-generation conjugate 2B (ABD-RM26 Gen 2B), features from generation 1 and generation 2A were combined. As in the first generation, RM26 was coupled to position 14 of ABD, but the DOTA chelator was introduced as a part of the N-terminus of the RM26 peptide, leaving both the C- and N-termini of ABD free. Similarly to generation 2A, a PEG_4_ linker was used as a spacer between the ABD and RM26 moieties.

The second-generation conjugate 2C (ABD-RM26 Gen 2C) resembles the Gen 2B design in terms of the position of RM26, but with a difference in the placement of the DOTA chelator. Instead of being coupled to the N-terminus of RM26, its position is changed to that of the C-terminus of ABD.

### 2.2 Production of ABD-RM26 Constructs

The ABD domain utilized in the production of second-generation ABD-RM26 conjugates was recombinantly produced by using a bacterial expression system. The purification of the crude sample was carried out by IMAC affinity chromatography. The final product, analyzed by SDS-PAGE (Figure S1) and MALDI-ToF (Figure S2), confirmed high purity and a molecular weight in concordance with the expected values (Table 2).

**Table 2:** Biophysical Characterization of ABD and Different ABD-RM26 Constructs^a^

construct	theoretical MW (Da)	experimental MW (Da)	*T*_m_ (°C)	*K*_D_, HSA (M)
ABD	7756.7	7759.4^b^	53.1	1.3 × 10^–11^
ABD-RM26 Gen 2A	8867.2	8870.6^c^	55.1	4.8 × 10^–11^
ABD-RM26 Gen 2B	9683.0	9681.9^c^	52.0	3.5 × 10^–11^
ABD-RM26 Gen 2C	9731.6	9731.1^c^	56.2	3.4 × 10^–11^

a MW: molecular weight; Tm: melting temperature; KD: equilibrium dissociation constant; HSA: human serum albumin.

b Analyzed by MALDI-MS.

c Analyzed by ESI-MS.

The chemical synthesis of the RM26 peptides was successfully performed using a fully automated SPPS system. The peptides were cleaved, ether-extracted, and purified with RP-HPLC. The purified RM26 probes were used as the starting material for the conjugation reaction with ABD. For Sortase A-mediated ligation of RM26 and ABD to generate the Gen 2A conjugate, we utilized a Sortase A variant (A3*). Gen 2B and 2C conjugates were the result of the cross-linking through the formation of an alkyl thioether between the thiol group of the Cys14 residue of ABD and chloroacetyl group of RM26. Following a final RP-HPLC purification step after the conjugation reactions, the purity and molecular weight of the final constructs were analyzed by analytical HPLC and ESI-MS (Figures S3 and S4). Experimental molecular weights showed values closely matching the theoretical expected molecular weight (Table 2) and purity was calculated to be over 90% for all conjugates.

### 2.3 Circular Dichroism Characterization of ABD-RM26 Conjugates

**Figure 2:**
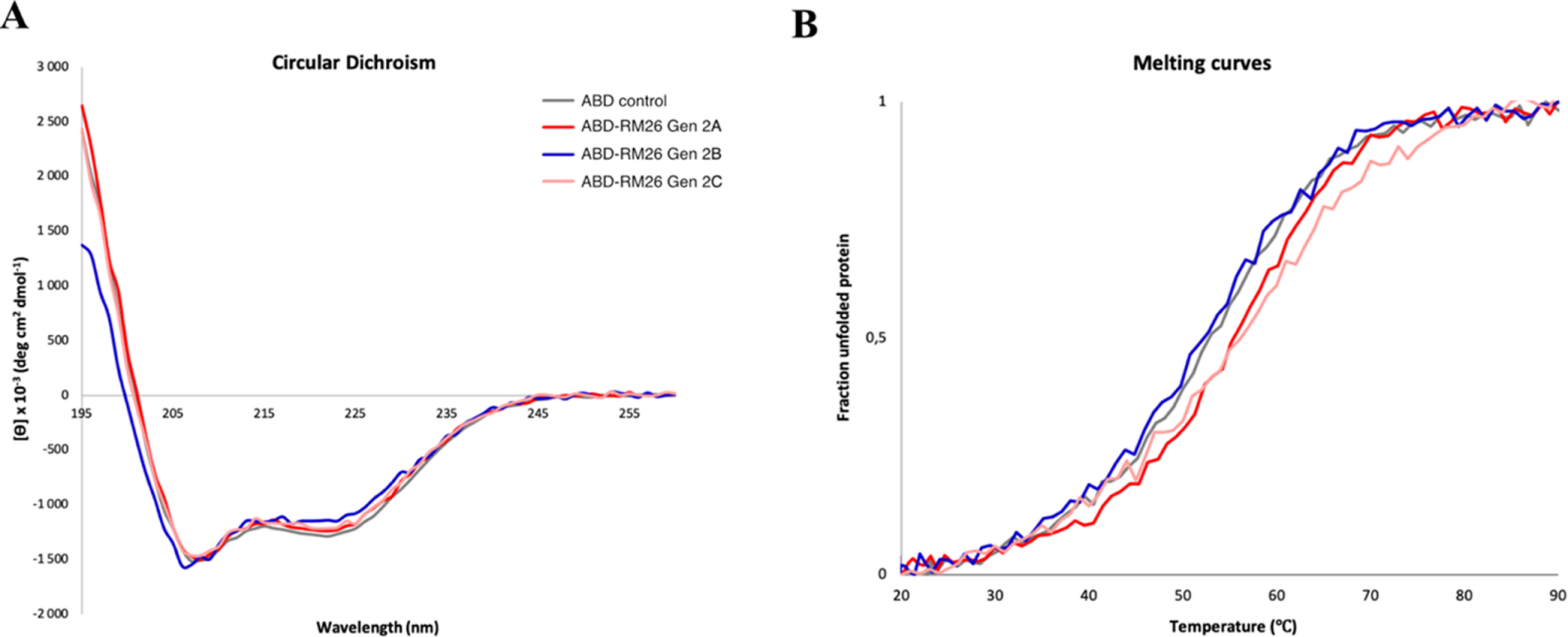
Circular dichroism (CD) spectroscopy analysis. (A) CD expressed in molar ellipticity for ABD control and ABD-RM26 conjugates. (B) Normalized melting curves of the ABD control and ABD-RM26 conjugates showing the fraction of unfolded protein as a function of temperature.

The secondary structure, thermal stability, and refolding of the ABD-RM26 conjugates were evaluated by circular dichroism (CD) spectroscopy. All conjugates exhibited the characteristic curve for α-helical confirmation with local minima at 208 and 222 nm (Figure 2A). Furthermore, a comparison of the CD spectra taken before and after denaturation confirmed the ability of all constructs to completely refold to their original secondary structures (curves not shown).

The helical content of the different constructs was estimated by calculation of the fraction helix. ABD control showed 47% helicity, while ABD-RM26 Gen 2A, 2B, and 2C displayed slightly lower values: 43%, 36%, and 40% helical content, respectively.

The thermal stability was studied by absorbance measurement at a wavelength of 221 nm during a temperature gradient. The melting curves displayed high-amplitude sigmoidal shapes with a calculated *T*_m_ ranging from 52 to 57 °C (Figure 2B and Table 2), which are similar to those of the unconjugated ABD control (53 °C). This shows that the presence of the RM26 peptide and the DOTA chelator has little effect on the thermal stability of ABD. Compared to the first-generation ABD-RM26 molecule reported earlier, the CD spectra and melting curves indicate a higher α-helical content and higher thermal stability for all of the second-generation designs.

### 2.4 Determination of the Binding Affinity to Human Serum Albumin

**Figure 3:**
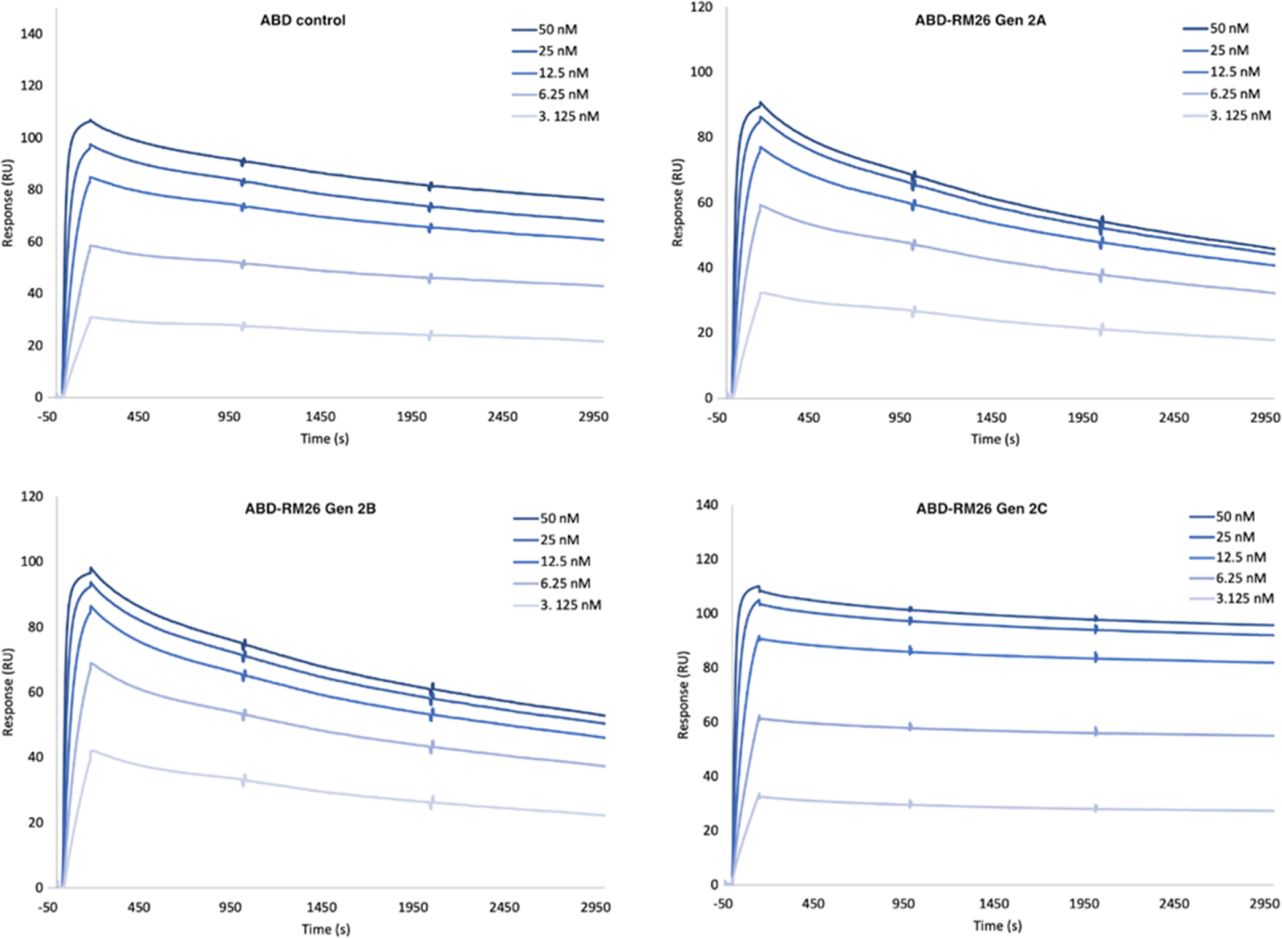
Surface plasmon resonance (SPR) sensorgrams of ABD-RM26 conjugates and the ABD control binding to surface-immobilized HSA. A 2-fold dilution series with five concentrations of each construct were injected onto an HSA surface. Affinities to HSA based on a Langmuir 1:1 curve fit are presented in Table S1.

To investigate the binding of the ABD domain of the conjugates to human serum albumin (HSA), the constructs were analyzed with a Biacore SPR instrument. HSA was immobilized on the surface of a CM5 gold sensor chip with the final immobilization level of 1250 RU. The sensorgrams obtained from the binding analysis showed concentration-dependent binding of all conjugates to HSA (Figure 3). Curves were fitted to the sensorgrams following the 1:1 Langmuir binding model in order to calculate the kinetic parameters (Table 2 and Table S1). The equilibrium dissociation constants (*K*_D_) for all constructs were calculated to be in the range of 10^–11^ M, indicating subnanomolar affinity to HSA.

### 2.5 Radiolabeling

The radiochemical yields for [^111^In]In-ABD-RM26 Gen 2A, [^111^In]In-ABD-RM26 Gen 2B, and [^111^In]In-ABD-RM26 Gen 2C were 82.2 ± 0.6, 41.4 ± 0.1, and 79 ± 1%, respectively. After NAP-5 purification, the radiochemical purities exceeded 95%. As much as 98–99% and 96–98% of activities were associated with intact labeled conjugates after incubation in PBS or blood plasma, respectively.

### 2.6 *In Vivo* Biodistribution

**Figure 4:**
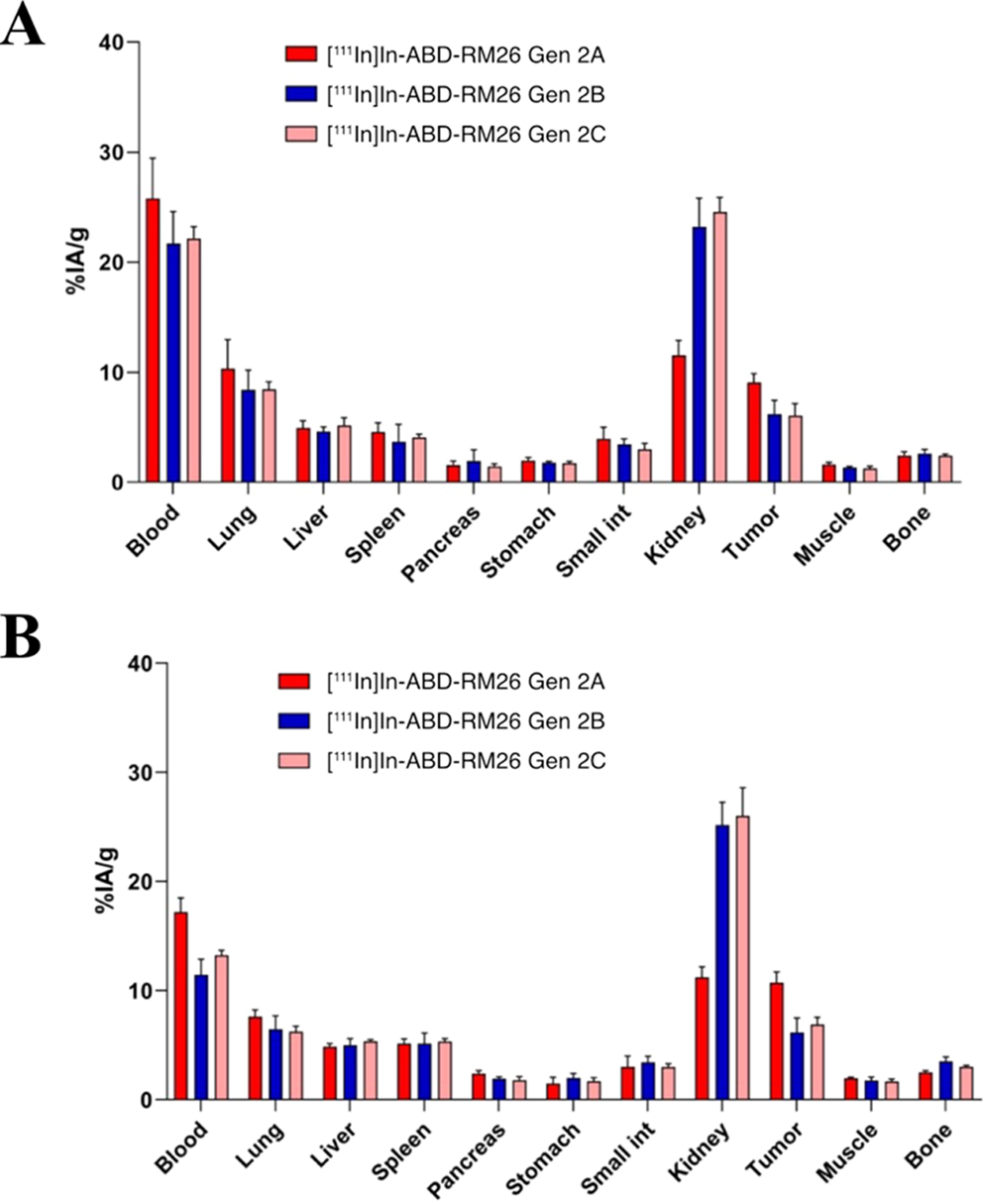
Biodistribution profiles of [^111^In]In-ABD-RM26 Gen 2A, [^111^In]In-ABD-RM26 Gen 2B, and [^111^In]In-ABD-RM26 Gen 2C at 4 h (A) and 24 h (B) post injection in Balb/c nu/nu mice bearing PC-3 xenografts. The bars represent the mean value and the error bars represent the standard deviation.

The biodistribution profiles of the three conjugates were similar (Figure 4 and Table S2). All conjugates had elevated activity concentration in blood at 4 h post injection, with the percentage of injected activity per gram (%IA/g) of 26 ± 4%IA/g for [^111^In]In-ABD-RM26 Gen 2A, 22 ± 3%IA/g for [^111^In]In-ABD-RM26 Gen 2B, and 22 ± 1%IA/g for [^111^In]In-ABD-RM26 Gen 2C. The activity concentration in blood decreased with time by 30–35% for [^111^In]In-ABD-RM26 Gen 2A and [^111^In]In-ABD-RM26 Gen 2C, and by 50% for [^111^In]In-ABD-RM26 Gen 2B; however, the activity concentration in blood was over 10% IA/g for all conjugates at 24 h pi. At this time point, the activity concentration in blood for [^111^In]In-ABD-RM26 Gen 2A was significantly higher than for the 2 other conjugates (*p* < 0.0001 for [^111^In]In-ABD-RM26 Gen 2B and *p* < 0.005 for [^111^In]In-ABD-RM26 Gen 2C).

The tumor uptake of [^111^In]In-ABD-RM26 Gen 2A at both studied time points was significantly (p value <0.015 for 2 h and <0.0015 for 24 h) higher (9.1 ± 0.8%IA/g) than for [^111^In]In-ABD-RM26 Gen 2B and [^111^In]In-ABD-RM26 Gen 2C (6 ± 1%IA/g and 6 ± 1%IA/g, respectively). At 24 h post injection, the tumor uptake did not change.

The renal excretion pathway was found for all three tested conjugates, which was manifested in the elevated activity uptake in kidneys together with low activity uptake in the liver and organs of the gastrointestinal tract (together with content). While activity uptake in liver was similar for all conjugates, activity uptake in kidneys differed statistically for [^111^In]In-ABD-RM26 Gen 2A (*p* < 0.0001). Activity uptake in kidneys did not change with time.

The only healthy tissue that demonstrated somewhat elevated activity uptake were the lungs at 4 h pi; however, activity uptake decreased with time, similarly to the activity concentration in blood.

**Figure 5:**
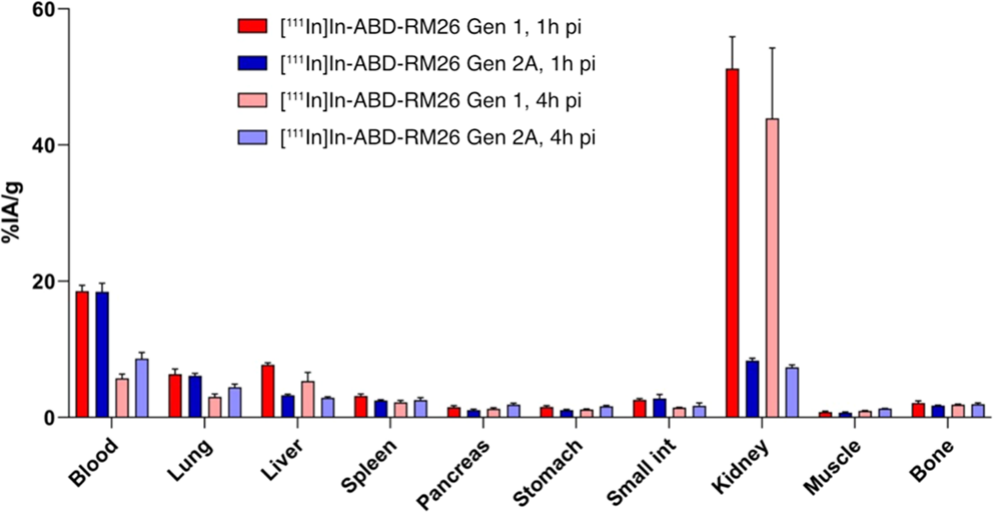
Biodistribution profiles of [^111^In]In-ABD-RM26 Gen 1 and [^111^In]In-ABD-RM26 Gen 2A at 1 and 2 h pi in NMRI mice. The bars represent the mean value and the error bars represent the standard deviation.

A direct comparison of the biodistribution of [^111^In]In-ABD-RM26 Gen 1 and [^111^In]In-ABD-RM26 Gen 2A in NMRI mice demonstrated that [^111^In]In-ABD-RM26 Gen 2A had a somewhat longer retention in blood circulation (Figure 5). The activity concentration in blood decreased 2-fold for [^111^In]In-ABD-RM26 Gen 2A, while for [^111^In]In-ABD-RM26 Gen 1 it decreased 3-fold. The most dramatic difference in biodistribution profiles was found in the activity uptake in kidneys; the activity uptake for [^111^In]In-ABD-RM26 Gen 1 was 6-fold higher. In other tested organs and tissues, the activity uptake and retention were similar for these two conjugates, except a 2-fold higher activity uptake in the liver for [^111^In]In-ABD-RM26 Gen 1 observed at both time points.

### 2.7 *In vitro* Characterization of [^111^In]In-ABD-RM26 Gen 2A

The conjugates were tested for binding specificity to GRPR in vitro by using PC-3 cells (Figure S4). Binding of the labeled conjugate [^111^In]In-ABD-RM26 Gen 2A decreased significantly (*p* < 0.05) when cells were pretreated with unlabeled GRPR-specific ligand, thus confirming the specificity of the new conjugate to GRPR. However, the specific binding of the labeled conjugate was low, 1.0–1.5% of added activity. Binding of [^111^In]In-ABD-RM26 Gen 2B and [^111^In]In-ABD-RM26 Gen 2C to GRPR-expressing cells was not inhibited by preincubation with the unlabeled ligand.

The kinetic data from the affinity measurements for [^111^In]In-ABD-RM26 Gen 2A performed in real time using living GRPR-expressing cells PC-3 demonstrated both a rapid association rate (*k*_a_ 6.7 ± 4.6 × 10^5^ M^–1^ s^–1^) and a rapid dissociation rate (*k*_d_ 1.3 ± 1.0 × 10^–2^ s^–1^). The calculated equilibrium dissociation constant (*K*_D_) was 120 ± 140 nM. The signal for specific binding of [^111^In]In-ABD-RM26 Gen 2B and [^111^In]In-ABD-RM26 Gen 2C was below detection level.

## 3 Discussion

Targeting GRPR using antagonistic bombesin analogues has demonstrated great potential for both radionuclide imaging and therapy of prostate cancer.^47^ The main challenge of the therapeutic application of short-peptide agents such as GRPR targeting RM26 is their short circulatory half-life. The fusion of the targeting peptide with an albumin-binding domain (ABD) is a promising strategy for half-life extension. By binding to human serum albumin (HSA) the size of the molecule complex increases, preventing kidney filtration as well as exploiting the neonatal Fc receptor (FcRn)-mediated recycling of albumin, thus prolonging the residence time in blood.

We previously reported that ABD-conjugated RM26 has an extended time in blood circulation compared to the peptide only; however, high uptake in healthy tissues such as the liver and kidney mitigated the use of this molecule for radionuclide therapy.^32^ This bispecific conjugate, ABD-RM26 Gen 1, demonstrated specific binding to both molecular targets, HSA and GRPR, and a high and very stable uptake in GRPR-expressing tumors. However, the assembly of three main building moieties in that conjugate was not optimal, which led to decreased helicity of the ABD protein. The low helicity of the ABD part resulted in its partial unfolding, which was identified as a main reason for high renal excretion of the conjugate.

The development of multifunctional conjugates (in this case, a bispecific conjugate suitable for stable labeling with a therapeutic radiometal) is an iterative process. In this follow-up study, we particularly aimed to develop a panel of second-generation ABD-fused RM26 peptides with a prolonged half-life, which could be utilized for radionuclide/drug/toxin therapy of GRPR-expressing tumors. Our hypothesis was that the molecular design of the conjugate would have an effect on its *in vivo* binding to albumin. To investigate this, we designed three new constructs with different formats and configurations of functional domains. The relative positions of the GRPR-binding moiety RM26 and the DOTA chelator to ABD were different in each construct, as depicted in Figure 1. All three second-generation conjugates shared a recombinantly produced ABD domain for half-life extension and a chemically synthesized RM26 peptide. The conjugation of ABD and RM26 was achieved following either enzymatic Sortase A-catalyzed ligation or chemical cross-linking, resulting in all cases in a semisynthetic molecule.

After the successful production of second-generation conjugates, biophysical characterization showed that they display excellent thermal stability. The measured melting temperatures were higher than for ABD-RM26 Gen 1 conjugate (50 °C) and closely matched the *T*_m_ of the unconjugated parental ABD035 molecule (Table 2). Circular dichroism spectroscopy was performed to analyze the secondary structures of the conjugates. After observation of the CD spectra, we confirmed that all constructs display curves characteristic of an α-helical structure, closely following the CD spectrum of a control ABD molecule. In contrast to our earlier study, where the 46-aa ABD was produced by SPPS,^32^ the recombinantly expressed ABD used here was produced with an N-terminal 6-aa peptide sequence, MEDANS. The importance of N-terminal residues for the interaction between bacterial albumin-binding domains and albumin has been reported earlier, leading us to explore an N-terminal extension for the protein.^48^ The sequence chosen for the ABD construct was a modified version of the N-terminal sequence used in the ABD-derived affinity proteins (ADAPT), a class of engineered scaffold proteins known to possess high thermal stability and favorable biodistribution properties when used for tumor targeting.^45,46^ We hypothesize that the peptide extension in our recombinant ABD construct could provide capping interactions that stabilize the helical structure. Helical content analysis of the constructs based on circular dichroism confirmed a higher relative helicity compared to the first-generation ABD-RM26 construct, where circular dichroism analysis showed only about 33% helicity (18 out of 56 residues in helical conformation).^32^ ABD-RM26 Gen 2A was 43% helical (34 out of 78 residues in helical conformation), Gen 2B was 36% (30 out of 84 residues in helical conformation), and Gen 2C was 40% (33 out of 84 residues in helical conformation). The lowest helicity was calculated for Gen 2B, which also had the lowest *T*_m_ (52 °C) of all of the new constructs. The data on helicity and thermal stability corroborate *in vivo* data for the first- and second-generation ABD-RM26 constructs (Figures 4 and 5). The [^111^In]In-ABD-RM26 Gen 2A construct, which had the highest helicity and thermal stability, demonstrated the best retention in blood circulation and the lowest kidney retention. Since the N- and C-terminal extensions and the RM26 peptide are not expected to be structured, the number of residues found in the helical conformation for the second-generation constructs correlates well with the data previously reported for the related albumin-binding domain G148-GA3 showing 80% helicity (37 out of 46 residues in helical confirmation) for the same region based on NMR structural data.^48^ From these results, we can conclude that a free N-terminus and an N-terminal peptide extension provided an ABD construct with a higher thermal stability and helicity. Furthermore, the helical structure of ABD was unaffected by conjugation to RM26. With these changes in molecular design, we were able to address the issues with the structurally less stable fully synthetic first-generation ABD-RM26.

The relative positions of RM26 and DOTA to ABD show a minimal impact on HSA binding, as shown in the SPR binding experiments (Table 2). The calculated K_D_ values for all conjugates were in the subnanomolar range, suggesting high-affinity binding to HSA. However, a small decrease in affinity could be observed compared to the unconjugated ABD control, mostly due to a higher dissociation rate (*k*_d_), resulting in faster dissociation. Surprisingly, the Gen 2C conjugate showed slower dissociation than the ABD control but overall lower affinity. Compared to the previously published first-generation ABD-RM26 conjugate with a *K*_D_ of 8.3 × 10^–11^ M, the second-generation conjugates showed a slightly higher affinity toward HSA.^32^ The small decrease in affinity compared to unconjugated ABD, with a *K*_D_ of 1.3 × 10^–11^ M, can be explained by the possible sterical interference of the RM26 peptide with HSA binding. Despite the small observed changes in affinity, we concluded that all of the constructs retained high affinity toward HSA and therefore could be used for evaluation *in vivo.*(32) The improved affinity toward HSA suggested a longer residence time in blood circulation.

The new-generation ABD-RM26 constructs were successfully and stably labeled with indium-111. Somewhat lower labeling yields were obtained for the ABD-RM26 Gen 2B construct, which might be explained by the possible sterical hindrance for the formation of a metal complex created by close connection to ABD and RM26 moieties. Previously it was demonstrated that coupling of the chelator either on the N-terminus or on helix 3 of the ABD domain does not influence either the ABD binding properties to albumin or loading of the chelator with the three-valent metal.^49^ Nevertheless, all constructs could be purified using a size exclusion column for an *in vivo* study using the PC-3 cell line xenograft mouse model. The biodistribution data for new conjugates showed that the ABD-RM26 Gen 2A conjugate had the longest circulation time in blood (Figure 4). At the same time, this conjugate demonstrated a significantly lower uptake in kidneys and a significantly higher uptake in tumors than the other two tested conjugates. These data corroborated the more stable helical structure and high thermal stability of this conjugate. However, the observed differences in biodistribution profiles of the three new conjugates, particularly in activity uptake in kidneys, did not correlate with their affinity to HSA (Table 2). While ABD-RM26 Gen 2C demonstrated a much slower off-rate than the other two conjugates and should theoretically have a lower fraction unbound to albumin in blood, this did not result in lower activity uptake in kidneys. We must admit that other factors, e.g., off-target interaction with other proteins present in the blood, could have a significant effect both on the residence time in blood circulation and on their absorption in excretory organs.^50^

The binding specificity to GRPR of the best-performing [^111^In]In-ABD-RM26 Gen 2A was further confirmed *in vitro* (Figure S4) and its biodistribution was compared head-to-head with the first generation of the ABD-RM26 conjugate (Figure 5). The comparison of the first generation of ABD-RM26 Gen 1 and ABD-RM26 Gen 2A conjugates was performed in NMRI mice (without xenografts), and therefore, their uptake in tumors was not compared. However, we can speculate that tumor uptake should not be better for the ABD-RM26 Gen 2A conjugate than for ABD-RM26 Gen 1, taking into account the weak affinity of the ABD-RM26 Gen 2A conjugate toward GRPR (120 nM). Unfortunately, the specific binding of [^111^In]In-ABD-RM26 Gen 2A was very low when tested *in vitro*, which was in agreement with its weak affinity to GRPR. It cannot be excluded that GRPR-mediated uptake of [^111^In]In-ABD-RM26 Gen 2A in tumors constitutes only part of the overall tumor uptake and the enhanced permeability and retention (EPR) effect of the albumin-ABD-RM26 adduct constitutes the rest. Two other studied conjugates, [^111^In]In-ABD-RM26 Gen 2B and [^111^In]In-ABD-RM26 Gen 2C, did not demonstrate any binding to GRPR *in vitro*. This result was not expected because ABD-RM26 Gen 1, which has a similar attachment of RM26 to the ABD domain, had an affinity for GRPR remaining in the nanomolar range. We have to conclude that the next step in development should be focused on improvement of binding to GRPR. On the other hand, the *in vivo* study corroborates that [^111^In]In-ABD-RM26 Gen 2A has better *in vivo* properties than [^111^In]In-ABD-RM26 Gen 1, which was manifested by the longer blood circulation of this conjugate and 6-fold lower kidney activity uptake. Taking into account the suboptimal affinity of ABD-RM26 Gen 2A for GRPR, we concluded that further *in vivo* characterization of this conjugate would be premature and unethical.

The main difference in the design of ABD-RM26 Gen 2A from other tested constructs was the linking of GRPR-targeting peptide RM26 to the C-terminus of the ABD scaffold, while position 14 was used for the attachment of the DOTA chelator. In all other constructs, position 14 was used for linking of the GRPR-targeting peptide RM26. We could speculate that DOTA chelator coupled to this position creates less sterical hindrance for binding to albumin *in vivo* than the bulkier peptide. The direct comparison of the biodistribution properties, e.g., retention in blood circulation and targeting properties, of the tested conjugates to other constructs of the ABD domain linked to small targeting peptides is not possible due to absence of the published data. The previously reported glucagon-like peptide 1 (GLP-1)-targeting conjugate GLP-1-ABD was not evaluated *in vivo*.^31^ Constructs of ABD fused with scaffold proteins (ADAPTs, affibody molecules, and DARPins, with molecular weights of 6–14 kDa) have been much more investigated. It is interesting that the binding properties to both targets (albumin and the secondary target of the scaffold protein) depend not only on the order of domain fusion but also on the scaffold’s target. It was reported that the HER2-targeting DARPin G3 demonstrated much better *in vivo* targeting properties when fused as G3-ABD than as ABD-G3,^51^ and the same effect, but to a lower degree, was observed for the HER2-targeting ADAPT-ABD and HER3-targeting affibody-based construct Z_HER3_-ABD.^37,43^ However, domain perturbation did not influence the targeting properties of the HER2-targeting affibody-containing constructs.^40^

Renal toxicity is an important limitation for the applicability of radiotracers to targeted radiotherapy. Literature data suggest that the absorbed dose for tumor should be at least 50 Gy to achieve therapeutic response, while the absorbed dose for kidney should not be higher than 23 Gy.^41^ The results of the present biodistribution study in tumor-bearing mice demonstrated that the tumor-to-kidney ratio desired for targeted therapy was not achieved, despite the dramatic improvement in pharmacokinetics for ABD-RM26 Gen 2A. Further modifications of the GRPR-targeting probes are required to enable their use for targeted therapy, including radiotherapy. A comparison of the ABD-RM26 Gen 2A conjugate’s residence time in blood with ABD-fused conjugates efficiently used in a preclinical model showed that toxicity to bone marrow should be acceptable in the precondition that tumor uptake will be improved at least 1.5–2-fold.^38,52^

In this study, we mainly focused on the function of ABD *in vivo*; the next step would be to improve the targeting properties of the GRPR-binding peptide. One approach could be to investigate factors that might improve peptide binding to GRPR, e.g., by introduction of a longer and more stringent spacer between ABD and RM26 or by inclusion of positively charged amino acids in the linker that improve the bombesin analogues’ affinity to GRPR.^53^ Another approach could be the use of a GRPR antagonist with better resistance to neutral endopeptidase, even though the results for ABD-RM26 Gen 1 suggested that the RM26 peptide moiety was resistant to neutral endopeptidase degradation in blood circulation.^22^ However, the position of RM26 in ABD-RM26 Gen 2A could result in its better exposure to neutral endopeptidase in blood circulation. Furthermore, the optimal choice of chelator for Lu-177 could also influence the overall biodistribution.^54^

## 4 Conclusions

In this study, we demonstrated that the molecular design of ABD-fused RM26 constructs has a significant impact on their biodistribution. Three second-generation conjugates were designed with different binding domain configurations, produced, and evaluated *in vivo*. All three new-generation conjugates containing the N-terminal 6-aa peptide MEDANS sequence displayed an improved biodistribution profile compared to the first generation. Additionally, the placement of the GRPR-binding antagonistic peptide in the C-terminus instead of position 14 on the ABD scaffold further decreased its reabsorption in the kidneys. The structural and thermal stability of the complex molecules proved to be an important factor contributing to the improved biodistribution. The Gen 2A ABD-RM26 conjugate displaying the best biodistribution profile can be a promising candidate for future engineering efforts and further development for radionuclide therapy of prostate cancer.

## 5 Methods and Materials

### 5.1 Recombinant Production of ABD

A plasmid containing the sequence of ABD035 with an N-terminal MEDANS amino acid motif, a Cys in position 14, and a C-terminal Sortase A recognition sequence followed by a His-tag was chemically transformed into *E. coli* BL21 (DE3)* cells. The recombinant protein production was carried out in tryptic soy broth with yeast extract (TBS-Y) media, and the culture was incubated at 37 °C until the optical density measured at 600 nm (OD_600_) reached 0.6–0.8, at which point it was induced by isopropyl β-D-1-thiogalactopyranose (IPTG) (Apollo Scientific, Bredbury, UK). After induction, protein production proceeded at room temperature (RT) overnight. The cells were harvested by centrifugation, and the pellet was resuspended in IMAC binding buffer (10 mM imidazole, 300 mM NaCl, 50 mM NaH_2_PO_4_) followed by sonication. The cell lysate was centrifuged and the supernatant was filtered and applied to a PD-10 column containing IMAC-resin (HisPur Cobalt resin, Thermo Fisher, Waltham, MA). The fractions containing the protein were pooled and buffer exchanged with Sortase A ligation buffer (50 mM Tris, 150 mM NaCl, 10 mM CaCl_2_) with a PD-10 desalting column (Cytiva, Uppsala, Sweden). The final purity and molecular weight of the protein were analyzed with sodium dodecyl sulfate polyacrylamide gel electrophoresis (SDS-PAGE) and matrix-assisted laser desorption ionization and time-of-flight analysis (MALDI-ToF) (MALDI TOF/TOF analyzer, Sciex, Applied Biosystems, Foster City, CA).

### 5.2 Synthesis of RM26 Peptides

All chemicals, amino acids, solvents, and synthesis reagents were purchased from Sigma-Aldrich (St. Louise, MO), Advanced Chematech (Louisville, KY), and NovaBiochem (St. Louise, MO) unless otherwise specified.

The RM26 peptides all shared a conserved nine amino acid sequence, responsible for receptor binding, but contained different N-terminal extensions and modifications due to the different conjugation strategies used for the production of the final molecule (Table 1). The peptides were chemically synthesized using an automated microwave-assisted solid-phase peptide synthesis (SPPS) instrument (Biotage Initiator Alstra, Uppsala, Sweden). Rink amide ChemMatrix resin (Biotage) with a loading capacity of 0.5 mmol/g was utilized as a solid support and the synthesis was performed on a scale of 0.1 mmol. Side-chain-protected amino acids were used for the synthesis following the 9-fluorenylmethyloxycarbonyl/*tert*-butyl (Fmoc/tBu) chemistry. Fmoc-statine was introduced with the 3-hydroxy group unprotected. The coupling step was carried out with diisopropylcarbodiimide (DIC)/Oxyma as the activation agent and deprotection was performed with 20% piperidine in *N*-methyl-2-pyrrolidone (NMP). Amino acids and coupling reagents dissolved in dimethylformamide (DMF) were added with 5 mol equiv.

The RM26 peptide constructs used for Gen 2B and 2C (Table 1) were synthesized with an N-terminal 4-methyltrityl (Mtt) side-chain-protected Lys residue for further modification. The Mtt group was selectively deprotected manually on resin using a 94:1:5 dichloromethane (DCM)/trifluoroacetic acid (TFA)/triisopropylsilane (TIS) reaction mixture. The peptide resin was incubated with the mixture for 2 min with shaking followed by removal of the deprotection reagent. This step was repeated (appr. ten times) until the completion of deprotection. The deprotected side chain was acetylated using chloroacetic acid. A reaction mixture containing 40 mol equiv of chloroacetic acid, 40 equiv of N,*N*′-dicyclohexylcarbodiimide (DCC), and 40 equiv of N,N-diisopropylethylamine (DIEA) dissolved in DCM was added to the resin and incubated at RT for 2 h.

The peptides were cleaved from the resin by incubation with a cleavage mixture containing 95:2.5:2.5 TFA/TIS/H_2_O for 3 h at RT. After cleavage, the mixture was added to 1:1 diethyl ether/H_2_O in order to extract the crude peptide from the cleaved side-chain-protecting groups. The crude peptide-containing water phase was separated and subsequently freeze-dried.

Crude peptides were purified by reversed-phase HPLC (RP-HPLC) (Agilent 1200 series, Agilent Technologies, Santa Clara, CA) on a semipreparative column (Zorbax 300SB-C18, 5 μm, 9.4 × 2500 mm, Agilent Technologies). The lyophilized peptides were reconstituted in buffer A (Milli-Q water +0.1% TFA) and injected into the column. A gradient of 20–50% of running buffer B (CH_3_CN + 0.1% TFA) was applied for a total running time of 30 min in order to separate the desired peptide from the side products. The fractions containing the final product were collected, pooled, and lyophilized for further use.

During the entirety of the synthesis procedure, coupling and deprotection steps were monitored with Ninhydrin test and the molecular weight of the cleaved peptides was confirmed with MALDI-ToF.

### 5.3 Production of ABD-RM26 Gen 2A

To produce ABD-RM26 Gen 2A, ABD and RM26 peptide were enzymatically cross-linked with Sortase A3* enzyme, carrying mutations P94S/D160N/D165A. This triple mutant displays significantly enhanced catalytic activity in comparison to wild-type enzyme.^55^ The recombinantly produced ABD containing a C-terminal Sortase A recognition motif (LPETG) was mixed with 2.5 molar excess of RM26 peptide-containing N-terminal Gly-Gly-Gly in Sortase A ligation buffer (50 mM Tris, 150 mM NaCl, 10 mM CaCl_2_) with the final concentrations of 100 and 250 μM, respectively. Lastly, 5 μM Sortase A3* enzyme was added and incubated at 37 °C for 1 h. The reaction mixture was purified with RP-HPLC using a similar protocol as described earlier. The fractions containing the final conjugates were collected and subsequently lyophilized.

After the cross-linking of ABD and RM26, a DOTA chelator was coupled to the conjugate by thiol-maleimide chemistry, utilizing Cys14 of ABD and maleimide-modified DOTA (Mal-DOTA) (MedChemExpress, NJ). The reaction was performed in phosphate-buffered saline (PBS, pH 7) by mixing ABD-RM26 with a 2-fold molar equivalent of Mal-DOTA followed by incubation at 55 °C for 2 h. The resulting conjugate was purified one more time with RP-HPLC as described above to ensure the high purity of the final product.

### 5.4 Production of ABD-RM26 Gen 2B

For the production of ABD-RM26 Gen 2B, a thioalkylation cross-linking reaction between the thiol group of ABD-Cys14 and the chloroacetyl group on the Lys of RM26 was performed. ABD was mixed with a 2-fold molar equivalent of RM26 in 60% PBS + 40% CH_3_CN, pH 8 conjugation buffer, and incubated overnight at 50 °C. The conjugation mixture was purified with RP-HPLC.

### 5.5 Production of ABD-RM26 Gen 2C

Cross-linking of ABD and RM26 was performed in the same manner as for ABD-RM26 Gen 2B. After the conjugation, the DOTA chelator was coupled to the conjugate by Sortase A-mediated enzymatic ligation. For this, a short peptide probe (GGGSSYGSK[DOTA]S) was synthesized chemically following the SPPS protocol described earlier, containing a DOTA-modified Lys amino acid and an N-terminal triple glycine sequence for Sortase A-mediated ligation. After ligation, the reaction mixture was purified with RP-HPLC.

The correct molecular weight of all conjugates was analyzed and confirmed with LC-MS electrospray-ionization mass spectrometry (ESI-MS) (LC - Thermo Ultimate3000, Thermo Fisher Scientific, Waltham, MA; MS - Bruker Impact II, Bruker Daltonics, Billerica, MA).

### 5.6 Circular Dichroism Characterization of ABD-RM26 Conjugates

For determination of the secondary structure and melting temperature of the different ABD-RM26 constructs, circular dichroism (CD) spectroscopy (Chirascan, Applied Photophysics, Leatherhead, U.K.) was used. Samples diluted to a final concentration of approximately 0.2 mg/mL (in 20 mM KH_2_PO_4_ buffer with 100 mM KCl pH 7.4) were subjected to the measurements. The CD spectra of the protein samples were obtained in the range of 195–260 nm at 20 °C.

To estimate the helical content of the different constructs, the measured ellipticity (Θ_obs_) was converted to mean residue ellipticity (MRE) using the formula MRE = Θ_obs_/10 × *l* × C × *n*, where *l* is the cuvette path length in cm, C is protein concentration, and *n* is the number of amino acids in each construct. Following this, fraction helix F_H_ was calculated using MRE at 222 nm (MRE_222_) using the formula *F*_H_ = (MRE_222_ – [Θ]_C_)/([Θ]_H_ – [Θ]_C_). Complete helix [Θ]_H_ and complete random coil [Θ]_C_ are given by [Θ]_H_ = −40 000 × ((1 – (2.5/*n*)) + 100 × *T* and [Θ]_C_ = 640–45 × *T*, where *T* is the temperature; in this case, 20 °C.^56^

The melting temperature curves were analyzed by heating the samples from 20 to 95 °C while monitoring the absorbance, and the melting temperatures were estimated from the transition point from folded to unfolded protein.

### 5.7 Surface Plasmon Resonance Experiments

In order to determine the affinity of the ABD-RM26 conjugates to human serum albumin (HSA), surface plasmon resonance analysis was carried out on a Biacore 8K system (Cytiva). HSA was immobilized on Series S CM5 type gold sensor chips (Cytiva) using standard amine coupling chemistry with *N*-hydroxysuccinimide/(1-ethyl-3-(3-dimethylamino) propyl carbodiimide hydrochloride (NHS/EDC) activating agents to a final level of 1250 RU. Unreacted NHS esters were deactivated with 1 M ethanolamine injected over the chip. One activated and deactivated blank surface was used as a reference. In all experiments, 5 concentrations (50 25, 12.5, 6.25, and 3.125 nM) of each construct were flown over the chip at a flow rate of 30 μL/min with PBS-T (PBS + 0.05% Tween-20, pH 7.4) running buffer. Association was allowed for 150 s followed by a 3000 s dissociation phase. Between concentrations, the surface was regenerated with the injection of 10 mM HCl solution for 30 s. The kinetic parameters were calculated with Biacore Insight Evaluation software.

### 5.8 Radiolabeling

The ABD-RM26 conjugates were radiolabeled with indium-111 as described previously.^22^ Briefly, indium-111 was added to 25 μg of each conjugate (0.54 MBq per 1 μg) in the presence of ammonium acetate buffer (pH 5.5). The reaction proceeded at 85 °C for 45 min. The radiochemical yield and purity were determined by instant thin-layer chromatography (iTLC (Agilent Technologies, Santa Clara, CA)) and RP-HPLC. iTLC was analyzed using a Cyclone Plus storage Phosphor System (PerkinElmer, Waltham, MA). Radio-HPLC analysis was performed using a Hitachi Chromaster HPLC system with a radioactivity detector and Phenomenex Luna C18 column (100 Å; 150 × 4.6 mm; 5 μm) at room temperature (20 °C). Solvent A was 0.1% trifluoroacetic acid (TFA) in H_2_O, solvent B was 0.1% TFA in acetonitrile, and the flow rate was 1 mL/min. The radiolabeled conjugates were purified by size exclusion using NAP-5 columns. To check the stability of the radiolabeled conjugates, 1 μg of purified labeled conjugate was incubated either in PBS at room temperature or in murine plasma at 37 °C for 2 h. The stability was determined by iTLC as described above.

### 5.9 *In Vivo* Biodistribution

All *in vivo* experiments were carried out on mice purchased from Scanbur A/S (Sollentuna, Sweden). All animal studies were approved by the Ethics Committee for Animal Research in Uppsala (Sweden), following the national legislation on the protection of laboratory animals (permit 5.8.18–11931/2020, approved 28 August 28, 2020).

The biodistribution profiles of [^111^In]In-ABD-RM26 Gen 2A, [^111^In]In-ABD-RM26 Gen 2B, and [^111^In]In-ABD-RM26 Gen 2C were compared in Balb/c nu/nu mice bearing PC-3 xenografts; the mice were implanted with 6 × 10^6^ PC-3 cells 4 weeks prior to the experiment (average mice weight 18 ± 1 g, tumor weight 0.12 ± 0.08 g, or tumor volume 0.11 ± 0.07 cm^3^). Each conjugate (30 kBq, 40 pmol) was injected intravenously and at predetermined time points of 4 and 24 h post injection, the mice (*n* = 4 animals per group) were euthanized after lethal intraperitoneal injection of ketamine/xylazine followed by exsanguination and the organs of interest were collected and measured for their radioactivity content using the γ counter.

The biodistribution profiles of [^111^In]In-ABD-RM26 Gen 1 and [^111^In]In-ABD-RM26 Gen 2A (30 kBq, 40 pmol) were compared head-to-head in NMRI mice 1 and 24 h pi (average weight 28 ± 2 g). At each time point, the mice (n = 4) were euthanized after lethal intraperitoneal injection of ketamine/xylazine followed by exsanguination, and the organs of interest were collected and measured for their radioactivity content using the γ counter.

### 5.10 In Vitro Characterization of [^111^In]In-ABD-RM26 Gen 2A

In vitro specificity test and measurements of real-time binding kinetics for new conjugates labeled with In-111 were performed using GRPR-expressing cells PC-3 using the methods described by Mitran et al.^57^ For experimental conditions, see Supporting Information.

### 5.11 Statistical Analysis

Statistical analysis was performed using GraphPad Prism 8.0 for Windows (GraphPad Software, San Diego, CA). An unpaired, two-tailed *t* test was assessed to determine the statistical significance between two groups and a one-way ANOVA with the Bonferroni test corrected for multiple comparisons was assessed to determine the statistical significance between more than two groups. The difference was considered as significant when the *P* value was less than 0.05.

## Supporting Information Available

Analysis of recombinantly produced ABD (SDS-PAGE and MALDI-ToF), biophysical characterization of second-generation ABD-RM26 conjugates ESI-MS analysis, kinetic parameters from surface plasmon resonance analysis of HSA binding, comparative biodistribution data of radiolabeled ABD-RM26 constructs, in vitro cell binding specificity test of ABD-RM26 Gen 2A (PDF)

## Abbreviations

GRPR: gastrin-releasing peptide receptor
ABD: albumin-binding domain
PSMA: prostate-specific membrane antigen
