# Supplementary material for: Evaluation of ABD-Linked RM26 Conjugates for GRPR-Targeted Drug Delivery
Source: ACS Omega. 2024 Aug 15;9(34):36122–33. doi: 10.1021/acsomega.4c00489 (PMC11359615; doi:10.1021/acsomega.4c00489)
Supplement: Supplementary file 1 — ao4c00489_si_001.pdf [file ao4c00489_si_001.pdf]

Evaluation of ABD-linked RM26 conjugates for GRPR-targeted drug delivery

Ábel Nagy[1,#], Ayman Abouzayed[2,#], Panagiotis Kanellopoulos[2], Fredrika Landmark[1], Ekaterina Bezverkhniaia[2,3], Vladimir Tolmachev[4], Anna Orlova[2,5], Amelie Eriksson Karlström[1,*]

1 Department of Protein Science, School of Engineering Sciences in Chemistry, Biotechnology and Health, KTH Royal Institute of Technology, AlbaNova University Center, 106 91 Stockholm, Sweden
2 Department of Medicinal Chemistry, Uppsala University, 752 37 Uppsala, Sweden
3 Research Centrum for Oncotheranostics, Research School of Chemistry and Applied Biomedical Sciences, Tomsk Polytechnic University, 634009 Tomsk, Russia
4 Department of Immunology, Genetics and Pathology, Uppsala University, 752 37 Uppsala, Sweden
5 Science for Life Laboratory, Uppsala University, 752 37 Uppsala, Sweden
# These authors contributed equally to this paper
* Correspondence: ameliek@kth.se

Supporting Information

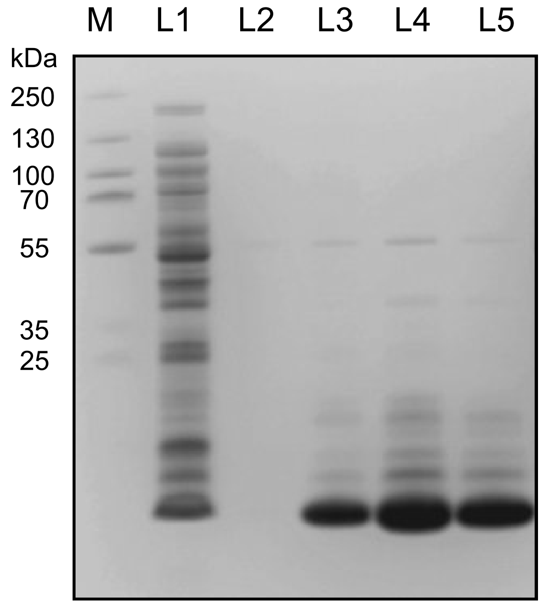

Figure S1. SDS-PAGE analysis of recombinantly produced ABD-Cys14. M: marker, L1: crude cell lysate, L2-L5: eluted fractions collected after IMAC purification.

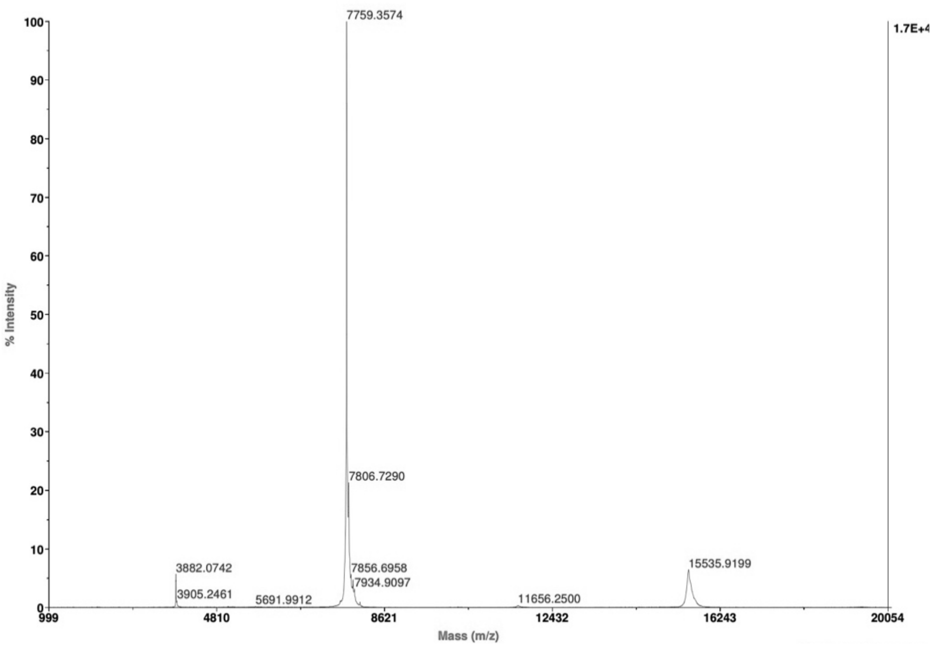

Figure S2. Determination of molecular weight (MW) of recombinantly produced ABD-Cys14 by MALDI-ToF. The measured MW (7759.4 Da) closely matches the theoretical weight (7756.7 Da).

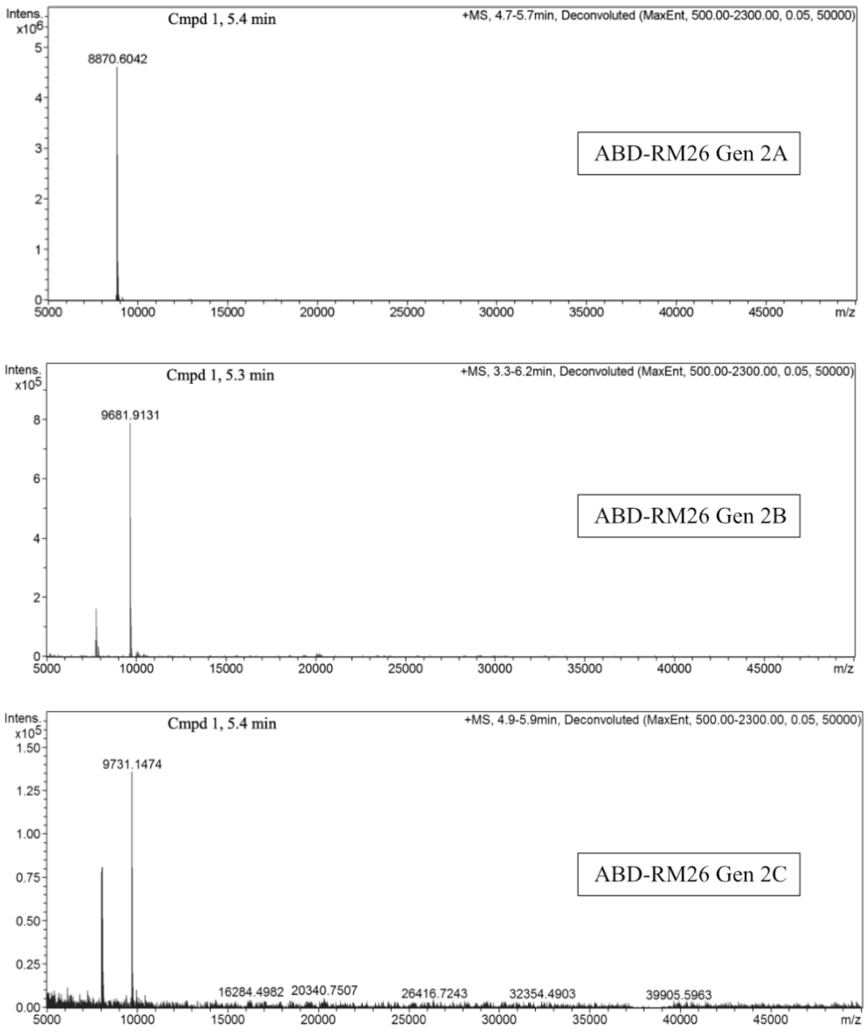

Figure S3. ESI-MS analysis of purified ABD-RM26 conjugates for molecular weight determination. The experimental molecular weight was compared with the theoretical molecular weight presented in Table 3. The impurity of lower molecular weight (7756.7 Da) appearing for Gen 2B and Gen 2C has been identified as the unconjugated MEDANS-ABD protein.

Table S1. Kinetic parameters and equilibrium dissociation constants (K_D) as determined by surface plasmon resonance (SPR).

**Construct**	**k_a (Ms^{-1})**	**k_d (s^{-1})**	**K_D (M)**
ABD-Cys14 control	1.8×10^{7}	2.4×10^{-4}	1.3×10^{-11}
ABD-RM26 Gen 2A	1.4×10^{7}	6.6×10^{-4}	4.8×10^{-11}
ABD-RM26 Gen 2B	2.3×10^{7}	8.0×10^{-4}	3.5×10^{-11}
ABD-RM26 Gen 2C	1.4×10^{6}	4.6×10^{-5}	3.4×10^{-11}

Table S2. Comparative biodistribution of ^{111}In-labelled ABD-RM26 in Balb/c nu/nu mice bearing PC-3 xenograft at 4 and 24 hours post-injection. The uptake is expressed as % ID/g and presented as an average value from 4 mice ± SD. One-way ANOVA with Bonferroni's multiple comparison test was performed to find significant differences.

**Organ or tissue**	**Uptake, % ID/g**		
	**ABD-RM26 Gen 2A**	**ABD-RM26 Gen 2B**	**ABD-RM26 Gen 2C**
**At 4 h post injection**			
Blood	26 ± 4.0	22 ± 3.0	22 ± 1.0
Lungs	10 ± 3.0	8 ± 2.0	8.5 ± 0.7
Liver	4.9 ± 0.7	4.6 ± 0.4	5.2 ± 0.7
Spleen	4.6 ± 0.8	4 ± 2.1	4.1 ± 0.3
Pancreas	1.6 ± 0.4	2 ± 1.0	1.5 ± 0.22
Stomach	2.0 ± 0.3	1.8 ± 0.11	1. 8 ± 0.2
Small Intestine	4.0 ± 1.0	3.5 ± 0.5	3.0 ± 0.5
Kidneys	12 ± 1.0	23 ± 3.0	25 ± 1.0
Tumor	9.1 ± 0.8	6 ± 1.0	6 ± 1.0
Muscle	1.6 ± 0.2	1.3 ± 0.1	1.2 ± 0.2
Bone	2.4 ± 0.4	2.6 ± 0.4	2.4 ± 0.2
GI	4.1 ± 0.4	3.7 ± 0.5	3.6 ± 0.4
Body	44 ± 2.0	38 ± 2.0	39 ± 2.0
	**ABD-RM26 Gen 2A**	**ABD-RM26 Gen 2B**	**ABD-RM26 Gen 2C**
**At 24 h post injection**			
Blood	17 ± 1.0	11 ± 1.0	13.2 ± 0.4
Lungs	7.6 ± 0.6	6 ± 1.0	6.2 ± 0.5
Liver	4.9 ± 0.3	5.0 ± 0.6	5.4 ± 0.1
Spleen	5.1 ± 0.4	5.1 ± 1.0	5.3 ± 0.3
Pancreas	2.4 ± 0.3	1.9 ± 0.2	1.8 ± 0.3
Stomach	1.5 ± 0.6	2.0 ± 0.4	1.7 ± 0.3
Small Intestine	3.0 ± 1.0	3.4 ± 0.6	3.0 ± 0.3
Kidneys	11.2 ± 1.0	25 ± 2.0	26 ± 3.0
Tumor	10.7 ± 1.0	6 ± 1.0	6.7 ± 0.7
Muscle	2.0 ± 0.1	1.8 ± 0.3	1.7 ± 0.2
Bone	2.5 ± 0.2	3.5 ± 0.4	3.0 ± 0.1
GI	2.82 ± 0.08	3.26 ± 0.07	3.2 ± 0.2
Body	45.6 ± 1.0	40 ± 3.0	41 ± 1.0

In vitro specificity test for [^{111}In]In-ABD-RM26 Gen 2 constructs

PC-3 cells (10^6 cells) were seeded on petri dishes one day before the experiment. One set of wells was pre-incubated with 1 μM/well of NOTA-PEG$_2$-RM26 to block GRPR. Thereafter, 1 nM/well of [^{111}In]In-ABD-RM26 Gen 2A was added to all wells and the dishes were incubated for 1 hour at 37 °C. The cells were then detached, collected and measured for their radioactivity content using the gamma counter.

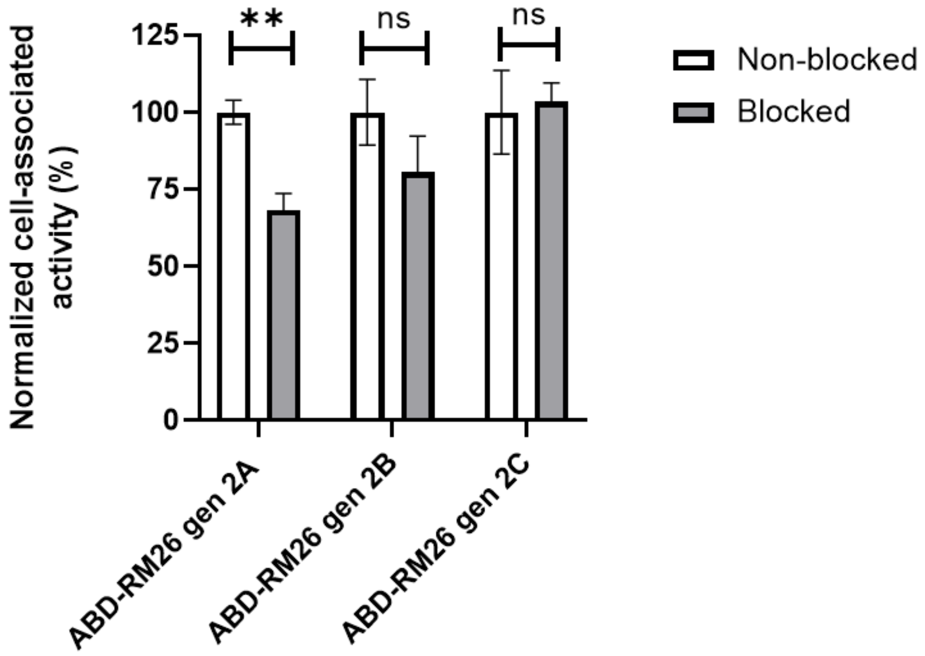

Figure S4. *In vitro* specificity test for [^{111}In]In-ABD-RM26 Gen 2 constructs on GRPR-expressing PC-3 cells.
